# Special Issue on Differential Gene Expression and Coexpression

**DOI:** 10.3390/biology12091226

**Published:** 2023-09-11

**Authors:** Vasileios L. Zogopoulos, Apostolos Malatras, Ioannis Michalopoulos

**Affiliations:** 1 Centre of Systems Biology, Biomedical Research Foundation, Academy of Athens, 11527 Athens, Greece; vzogopoulos@bioacademy.gr; 2 Section of Cell Biology and Biophysics, Department of Biology, National and Kapodistrian University of Athens, 15701 Athens, Greece; 3 Biobank.cy Centre of Excellence in Biobanking and Biomedical Research, University of Cyprus, 2029 Nicosia, Cyprus

The most common approach in transcriptomics (RNA-seq and microarrays) is differential gene expression analysis (DGEA). Differentially expressed genes (DEGs) may be responsible for phenotypic differences between various biological conditions. An alternative approach is gene coexpression analysis, which detects groups of genes with similar expression patterns across unrelated sets of transcriptomic data from the same organism. Coexpressed genes tend to be involved in similar biological processes. This Special Issue includes 12 research articles and one review on the topic of differential gene expression and coexpression. This review is an introduction to the basic methods of coexpression analysis, while the research articles describe both software and tools that assist in the execution of differential gene expression and coexpression analysis, as well as computational workflows that reveal new biological knowledge.

Through a microarray-based comparison of *dcap-1* knockout and wild type roundworms, Borbolis et al. [1] revealed the role of mRNA DeCAPping enzyme (*dcap-1*) in the silencing of spermatogenic genes during late oogenesis and in the suppression of aberrant immune gene rise during aging in *Caenorhabditis elegans*. Yoon et al. [2] revealed that high levels of procollagen C-endopeptidase enhancer 2 (PCOLCE2) in Tonsil-Derived Mesenchymal Stem Cells (TMSCs) were highly effective in potentiating ROS generation in co-cultured differentiated HL-60 (dHL-60) cells through an RNA-seq-based DGEA. By performing CAGE-seq on two types of atrophic rat muscle samples and through a bioinformatics pipeline centred on Transcription Start Site (TSS) analysis, Pintus et al. [3] identified non-coding transcribed regulatory elements controlling the skeletal muscle disuse-mediated atrophy. Otálora-Otálora et al. [4] used a bioinformatics workflow which includes differential expression, and coexpression network and coregulatory network analyses of microarray samples to discover possible unique lung cancer biomarkers, as well as possible common biomarkers in tumours (lung, breast, and leukaemia). Through an integrative bioinformatics pipeline, including the DGEA of hepatocellular carcinoma and normal liver microarray samples, and Weighted Gene Coexpression Network Analysis (WGCNA), Nguyen et al. [5] identified five hub genes as potential prognostic biomarkers in liver cancer. Singh et al. [6] were able to identify four Oral Squamous Cell Carcinoma (OSCC)-specific hub genes, using a bioinformatics workflow including a DGEA of selected RNA-seq samples from TCGA, the construction of a weighted protein–protein interaction network and, through module discovery, the identification of a single module of 12 genes as significant. Through a pipeline composed of expression stability and a subsequent quantitative PCR analysis of reference genes, Unkovič et al. [7] showed that archived formalin-fixed and paraffin-embedded (FFPE) colon samples of DSS-induced colitis mouse samples are a reliable source of RNA. Pyatnitskiy et al. [8] assessed the Oxford Nanopore Technologies (ONT) Long-read Direct RNA-seq as a platform suitable for the estimation of gene abundance by comparing an ONT analysis pipeline with an Illumina RNA-seq-based one. Their results showed that at least 2 ONT replicates are needed to detect expressions of most genes and transcripts. Zogopoulos et al. [9] offered a review on all available types of gene coexpression analysis using transcriptomics data, as well as a presentation of the most popular web and standalone gene coexpression software tools. They also listed additional downstream analyses, including biological term enrichment analysis tools. One such tool is FLAME [10], a web app developed to simplify handling multiple gene lists during functional enrichment analysis. It enables intersections and unions among gene lists, with results displayed interactively via heatmaps, bar charts, Manhattan plots, and networks. BioTEA [11] is a Linux command line tool that performs microarray-based DGEAs. BioTEA facilitates data retrieval from microarray studies as well as the automatic parsing of their metadata, followed by differential analysis steps using simple commands up to the production of DEG lists, which could then be used for subsequent analysis. ARGEOS [12] is a web tool to streamline dataset discovery from GEO and ArrayExpress public transcriptomics repositories while also offering in-depth dataset analysis, encompassing protocol collection, dataset count, and supplementary reference details. Finally, MAGE [13] is an open-source tool for gene expression data meta-analysis. It features probe-to-gene conversion, standard analysis and meta-analysis with bootstrap errors, multi-outcome meta-analysis, and functional enrichment. Comprehensive visualisations accompany each function while standalone and webserver versions of MAGE are offered.

Τhe guest editors are grateful to all authors for their contributions to this Special Issue. Their works enlighten various aspects of differential gene expression and coexpression analyses, starting from transcriptomics data production and automated data acquisition from public repositories, followed by comprehensive analysis and meta-analysis applications and downstream analysis tools. Some publications presented bioinformatics pipelines used for the discovery of cancer biomarkers. We are looking forward to the presentation of novel computational pipelines and biological discoveries in the Special Issue “Differential Gene Expression and Coexpression 2.0”.
